# Acoustic evaluation of behavioral states predicted from GPS tracking: a case study of a marine fishing bat

**DOI:** 10.1186/s40462-019-0163-7

**Published:** 2019-06-14

**Authors:** Edward Hurme, Eliezer Gurarie, Stefan Greif, L. Gerardo Herrera M., José Juan Flores-Martínez, Gerald S. Wilkinson, Yossi Yovel

**Affiliations:** 1 Department of Biology, University of Maryland, College Park, MD 20742 USA; 2 School of Zoology, Faculty of Life Sciences, Tel-Aviv University, 6997801 Tel-Aviv, Israel; 3 Sagol School of Neuroscience, Tel-Aviv University, 6997801 Tel-Aviv, Israel; 4 Estación de Biología de Chamela, Instituto de Biología, Universidad Nacional Autónoma de México, 48980 San Patricio, Mexico; 5 Laboratorio de Sistemas de Información Geográfica, Departamento de Zoología, Instituto de Biología, Universidad Nacional Autónoma de México, 04510 Ciudad de México, Mexico

**Keywords:** Behavioral change point analysis, Correlated velocity movement, Expectation maximization and binary clustering, First-passage time, Foraging, GPS telemetry, Hidden Markov models, K-means, Path segmentation

## Abstract

**Background:**

Multiple methods have been developed to infer behavioral states from animal movement data, but rarely has their accuracy been assessed from independent evidence, especially for location data sampled with high temporal resolution. Here we evaluate the performance of behavioral segmentation methods using acoustic recordings that monitor prey capture attempts.

**Methods:**

We recorded GPS locations and ultrasonic audio during the foraging trips of 11 Mexican fish-eating bats, *Myotis vivesi*, using miniature bio-loggers. We then applied five different segmentation algorithms (k-means clustering, expectation-maximization and binary clustering, first-passage time, hidden Markov models, and correlated velocity change point analysis) to infer two behavioral states, foraging and commuting, from the GPS data. To evaluate the inference, we independently identified characteristic patterns of biosonar calls (“feeding buzzes”) that occur during foraging in the audio recordings. We then compared segmentation methods on how well they correctly identified the two behaviors and if their estimates of foraging movement parameters matched those for locations with buzzes.

**Results:**

While the five methods differed in the median percentage of buzzes occurring during predicted foraging events, or true positive rate (44–75%), a two-state hidden Markov model had the highest median balanced accuracy (67%). Hidden Markov models and first-passage time predicted foraging flight speeds and turn angles similar to those measured at locations with feeding buzzes and did not differ in the number or duration of predicted foraging events.

**Conclusion:**

The hidden Markov model method performed best at identifying fish-eating bat foraging segments; however, first-passage time was not significantly different and gave similar parameter estimates. This is the first attempt to evaluate segmentation methodologies in echolocating bats and provides an evaluation framework that can be used on other species.

**Electronic supplementary material:**

The online version of this article (10.1186/s40462-019-0163-7) contains supplementary material, which is available to authorized users.

## Background

Animal movement data are becoming increasingly abundant for marine and terrestrial vertebrate species at ever finer spatial and temporal resolutions, allowing researchers to address a variety of ecological questions from the point of view of the individual [1–3]. However, inferring what an animal is doing from complex movements can be challenging given that different behaviors may exhibit similar movement features. For example, multiple GPS locations concentrated in a small area might indicate short tortuous movements, occurring when an animal searches for prey, or simply a resting animal with poor signal quality causing random fluctuations [4]. Distinguishing between these alternatives is sometimes possible based on an analysis of multiple movement features. For example, an animal foraging on a productive patch often moves more slowly while turning frequently. Such area-restricted search (ARS) behavior has been predicted from optimal foraging theory and observed in several animals that exploit patchy resources [5, 6].

This example illustrates the basic approach underlying path segmentation methods, which detect patterns in movement to provide insight into underlying behavioral states and partition tracks into segments of distinct behavioral states [7, 8]. These methods typically fall into three categories: pattern description, process identification, or change point detection [7]. *Pattern description* methods involve estimation of primary movement parameters, such as speed and turn angle, or secondary parameters derived from windows of many steps [7]. Parsing locations into distinct behaviors can be accomplished through simple thresholding schemes that manually separate short- and long-range movements or through unsupervised clustering, such as k-means clustering (kmC) [9, 10]. First-passage time (FPT), a commonly used secondary parameter, calculates the time to enter and leave a virtual circle for every location on an animal track, and is often used for revealing locations of intensive search [11]. Alternatively, *process identification* methods infer behaviors that shape the movement data [12, 13]. These methods model the change in speed and turn angle through time and space to annotate the animal’s movement with behavioral states. For example, Hidden Markov models (HMM) estimate a sequence of predefined states as well as the switching probabilities between states [12, 13]. Expectation-maximization and binary clustering (EMbC) is a simple alternative state-space model that sequentially groups locations into clusters of high and low velocity and turn angle [14]. Finally, *change point detection* methods use a moving window to examine portions of the path to determine where local means differ from global averages of movement parameters under the assumption that these locations indicate switches in behavioral states [7, 8, 15]. Correlated velocity movement (CVM) models use continuous time to allow for irregularly sampled data and can be combined with change point detection (CVCP) [16].

Despite growing interest in making inferences about behavioral states from movement patterns [17], methods are rarely validated in wild animals that are difficult to directly observe [10, 18]. Exceptions include records of foraging events captured by sensors on-board large animals, such as cameras on gannets [19, 20], time-depth recorders on elephant seals [21], stomach temperature loggers in tuna [22], or accelerometers on monk seals [23], all of which provide independent validation of behavioral states.

Recent miniaturization of biologgers allows integration of ultrasonic microphones with GPS tracking to record movement and vocalizations of bats [24–28]. Many bat species use echoes from ultrasonic vocalizations to detect obstacles and prey while foraging [29, 30]. These biosonar calls dynamically change in inter-pulse interval (IPI), frequency, and duration depending on the environment and behavioral context [31–33]. Echolocating bats that capture prey while flying emit a feeding buzz - a characteristic sequence of calls that decrease in IPI, frequency, and duration - when approaching and attempting to capture a prey item [29, 34]. Feeding buzzes provide a reliable cue that indicates foraging behavior.

**Fig. 1:**
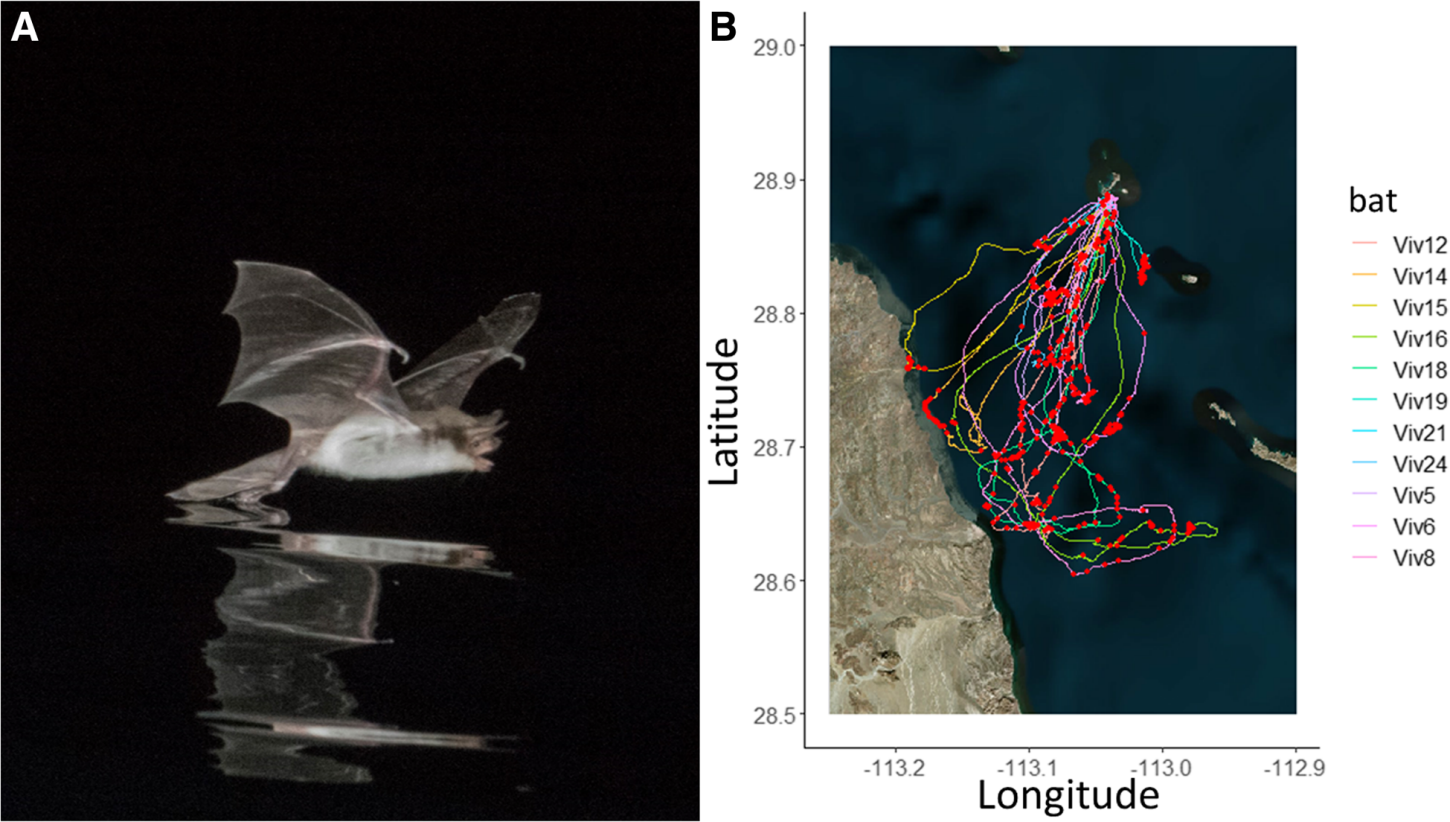
**a** Photo of a Mexican fish-eating bat (*Myotis vivesi*) trawling for prey and (**b**) a satellite map (“Esri.WorldImagery”) of the study area with GPS tracks of each foraging trip overlaid. These bats use biosonar to sense their environment, such as the ocean surface, and cue into small prey that might break the surface. Prey capture attempts, or feeding buzzes, recorded from an on-board ultrasonic microphone are overlaid on each trip. (Photo credit: Glenn Thompson)

We used acoustic and GPS biologgers to investigate the foraging behavior of the Mexican fish-eating bat (*Myotis vivesi*, Menegaux, 1901; henceforth “fish-eating bat”), which is endemic to the islands and coasts of the Gulf of California, Mexico [35]. *M. vivesi* eat predominantly small marine crustaceans and larval fish captured from the surface of the ocean (L.G. Herrera M., and E. Claire, personal communication; Fig. 1) [36]. Piscivorous bats independently evolved in two bat genera, *Noctilio* and *Myotis* [37]. Piscivorous bats in the genus *Myotis*, like our study species, use biosonar to detect prey that break the water surface and use feeding buzzes when capturing prey with targeted dips of their hindfeet [37–40] (Fig. 1). GPS tracking revealed that these bats often travel over 20 km to forage each night as they search for unpredictable patches of prey [26]. These foraging trips often contain over a dozen short foraging bouts (9 min average) [26].

In this study, we apply and compare five segmentation methods (Table 1) that include pattern description (k-means clustering and first-passage time), process identification (hidden Markov models and expectation-maximization and binary clustering), and change-point detection with behavioral partitioning (correlated velocity change point analysis) to predict foraging behavior in fish-eating bat foraging trips. We use feeding buzzes in audio recordings on-board free-flying bats to confirm foraging, and then evaluate the performance of each of the methods mentioned above using true positive rate (buzzes in predicted foraging locations), true negative rate (absence of buzzes in predicted commute locations), and balanced accuracy (the average of true positive and true negative rates). To distinguish methods with similar balanced accuracy, we also compare speed and turn angles predicted for foraging by each method against speed and turn angles at locations containing feeding buzzes.

**Table 1:** Tuned parameters and settings for each of the five segmentation methodologies

Category	Method	Parameter	Setting
Pattern Description	k-Means clustering	–	–
	First-passage time	Radius	250 m
		Threshold	142 s
State-space modeling	Expectation Maximization and binary clustering	–	–
	Hidden Markov model	Regularization	15 s
		Initial step length (mean/SD)	State 1: 70.2/ 27.6 m
			State 2: 160.8/ 23.0 m
		Initial turn angle (mean/concentration)	State 1: 0/0.1
			State 2: 0/0.1
Behavioral Change Point Analysis	Correlated velocity movement behavioral partitioning	Window size	2 (2.5)^a^ min
		Window step	1.25 min
		Minimum changepoint distance	0.5 min

^a^One bat flight did not converge with a 2 min window size and was adjusted to 2.5 min

While it remains unclear how fish-eating bats decide where to initiate hunting, a high density of fish at the water surface can trigger trawling in other fishing bats [39]. Direct observations and preliminary movement analyses suggest that *M. vivesi*, like many other marine predators searching for unpredictable patches of prey [41, 42], use ARS when foraging and fast straight movement when commuting [26]. Therefore, we expected more feeding buzzes in areas of the trip indicative of ARS than when the animal was traveling between patches or returning to the roost. Our goals were to determine the best performing methods for this data set and provide a framework for researchers to use with independent behavioral data to evaluate segmentation methods.

## Methods

### Data collection

We conducted the study on Isla Partida Norte (28^°^ 53′ 16″ N, 113 ^°^ 02′ 30″ W), a 1.4-km^2^ island located in the midriff region of the Gulf of California, Mexico [43]. The island holds the largest known colony of *M. vivesi* (~ 8000 individuals) [44]. Our study was conducted between May 27 and June 19, 2015, at which time females are nursing pups (permits #7668–15 and 2492–17 from Dirección General de Vida Silvestre, and permits #17–16 and 21–17 from Secretaría de Gobernación, and the University of Maryland Institutional Animal Care and Use Committee protocol FR-15-10).

Bats were captured by hand in the morning from under rocks on tallus slopes along the south-east region of the island. Lactating females weighing 32.5 ± 2.8 g (mean ± SD) were selected for tagging to facilitate recapture when the bats returned to feed their pups during the day. We glued biologger tags (Robin GPS Loggers, CellGuide Ltd., Israel) with 8 GB of memory and VHF radiotransmitters (Holohil BD-2X) weighing on average 4.6 ± 0.1 g (mean ± SD) to the back of each bat using non-toxic glue (Perma-Type Surgical Cement, Plainville, Connecticut) [24]. We released bats at their capture locations during midday. After 1 or 2 days, we used the transmitters to locate and recapture tagged bats. Because our tags exceed conventional recommended weight allowances for tags (see [45] for review), we conducted a series of trials to determine the effect of the tags on the bats (see also [26]). First, we confirmed that bats with a tag could fly and forage normally in a flight tent with a pool [26]. We then compared trips for bats with GPS tags against bats carrying 0.5 g telemetry tags and found no difference in duration of trips (telemetry: 4.3 ± 2.1 h, GPS: 3.8 ± 1.8 h; *p* = 0.4, permutation t-test *N* = 20 GPS, *N* = 15 telemetry) and no difference in weight loss between telemetry and GPS-tagged bats after controlling for number of days tagged (ANCOVA: F_1,63_ = 1.55, *p* = 0.22, *N* = 47 GPS, N = 20 telemetry). We also found no difference in condition (weight to forearm ratio) of pups whose mothers were GPS or telemetry-tagged (ANCOVA: F_1,5_ = 2.31, *p* = 0.20, *N* = 5 GPS, *N* = 3 telemetry). Finally, we confirmed that conspecific vocalizations were present throughout GPS-tagged bat trips [26], indicating that bats with GPS tags traveled to the same foraging areas as bats without GPS tags.

**Fig. 2:**
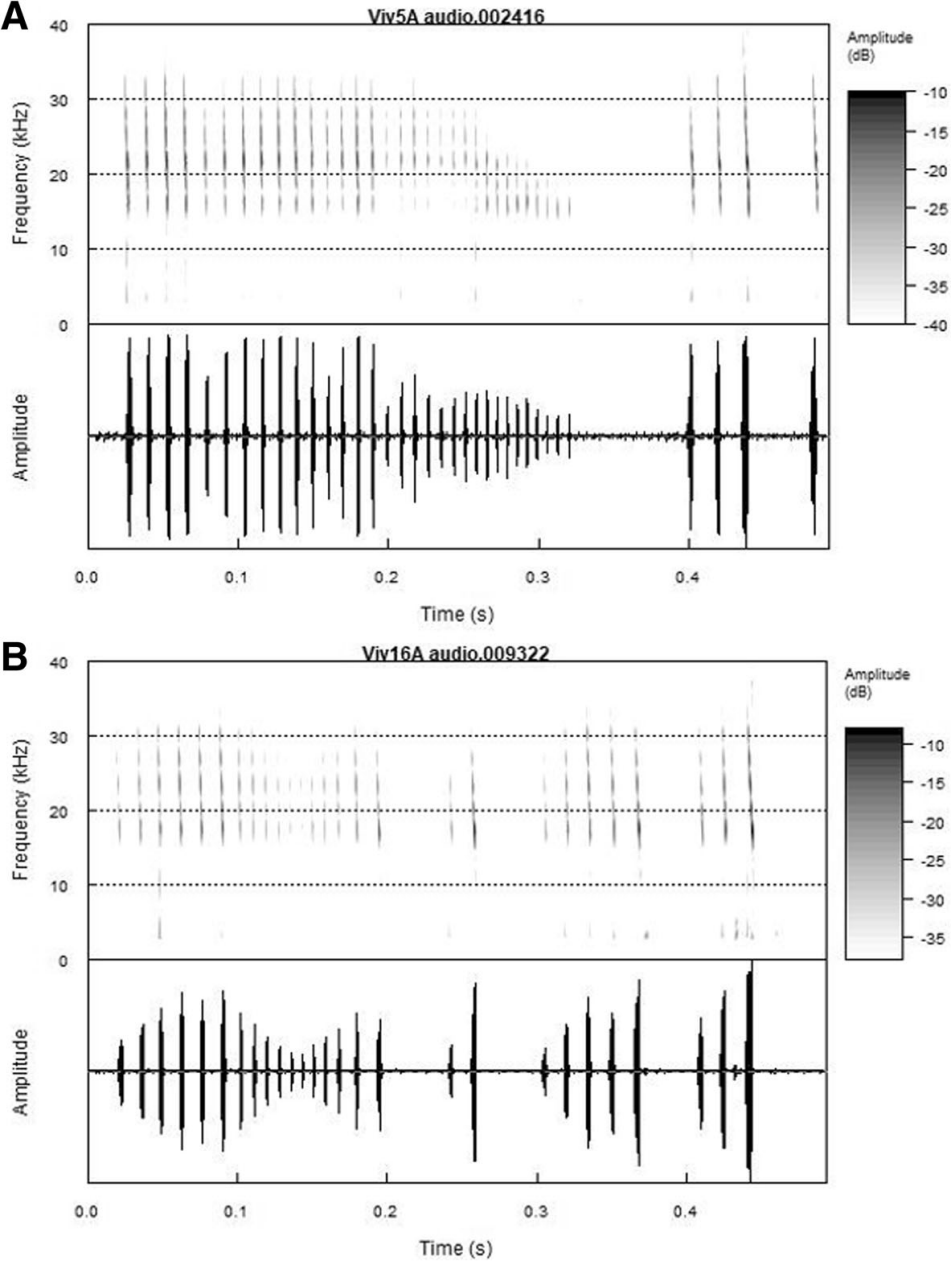
Spectrogram (top) and waveform (bottom) of fish-eating bat echolocation calls. (**a**) Typical terminal buzz, (**b**) aborted buzz (ends at 0.2 s) followed by search phase calls

During the night, tags recorded GPS locations every 15 s and 0.5 s of audio every 5 s (10% duty cycle at 184 kHz sampling rate). While searching for prey, on-board audio reveals that bats typically emit calls with a duration of 6 ms and with intervals of 200 ms between calls, consistent with prior call measurements [46]. When approaching prey, the echolocation calls and intervals become progressively shorter and terminate in a feeding buzz. A feeding buzz lasts 0.2–0.25 s (Fig. 2); thus, a 0.5 s recording is sufficient to distinguish search phase calls from a buzz. Foraging bouts typically last 6 min (see Results) with dozens of attacks performed during each bout. Therefore, a 10% duty cycle with a recording every 5 s almost always captures attacks in a foraging bout. To validate this assertion, in 2017 we tagged three bats with new tags (Vesper, ASC. Inc.; Additional file 1: Table S2), which allow continuous audio recordings (one 50% and two 100% duty cycle) for an entire night. We then sub-sampled those recordings to mimic the 10% duty cycle data to determine how often a 10% sampling rate resulted in missing foraging bouts (we performed all possible shifts of 0.5 s out of 5 s). The analysis showed that a 10% sample rate detected 77 ± 16% of foraging bouts (mean ± SD, Additional file 1: Figure S2).

### Analysis of on-board audio

On-board audio was analyzed using custom Matlab software called “Batalef,” [25]. Calls were identified with a peak detection algorithm and manually checked. Call start and end points were determined using a 5 dB drop from the peak amplitude. Inter-pulse interval (IPI) was measured as the time between the end of one call and the start of the next. We identified buzzes by searching for calls below an IPI threshold [47] of 10 ms that occurred in groups of at least three consecutive calls (Fig. 2). This was detected automatically and then validated by manual inspection. We included sequences with a terminal buzz as well as instances of aborted buzzes as both suggest that the bat was engaged in foraging behavior and attempting to capture prey [34]. Because the biologger sampling schedule creates three audio recordings (before, during or after) for each GPS location, we aggregated buzzes detected in the audio files closest to a GPS fix to determine the location and movement characteristics for each buzz (Fig. 1b).

### Analysis of location data

GPS and audio tags were deployed on eleven bats over a 10-day period resulting in fifteen trips (Additional file 1: Table S1). All locations were transformed to Cartesian coordinates using a Universal Transverse Mercator (UTM) 12 N projection. GPS accuracy is ca. 8 m in the X-Y plane and ca. 11 m in the Z axis [25]. We excluded all GPS locations within 250 m of the island and subsequent tracks with fewer than 100 locations to remove readings while bats were in the roost or making short movements around the island. Speed, distance, and absolute value of turn angle (hereafter “turn angle”) between subsequent steps were then calculated for each pair of successive locations along the path of each bat trip. Paths were segmented into either foraging or commuting states using five segmentation methods [7]. Below we describe how we estimated parameters associated with each of the five methods.

### K-means clustering

K-means clustering (kmC) takes a set of *n* data points to be clustered into *k* clusters and finds a partition that minimizes the squared error between the mean of a cluster and the points in that cluster [48]. We applied kmC to speed and turn angles using the “kmeans” function in the R package “stats” [49]. To determine the optimal number of clusters, we investigated the percentage of variance explained by kmC over a range of cluster sizes to find an “elbow,” or the location at which adding more clusters only marginally increased variance explained [50]. The elbow method showed that two clusters was the optimal number of partitions. We labeled the cluster with the lowest speed and highest turn angle as foraging [10]. After overlaying behavioral states on the parameter values, clustering produced a linear threshold at a turn angle of 75° (Additional file 1: Figure S3).

### First-passage time

First-passage time (FPT) is the time it takes for an individual to enter and leave a virtual circle of fixed radius drawn around each location [51]. High FPT values are generally associated with slower and more tortuous movements, such as area-restricted searching, while low FPT values are generally associated with faster and more straight-line movements such as commuting. We used the R package “adeHabitatLT” to calculate FPT for all tracks [11, 52]. To determine a common scale to compare FPT values between individuals, we calculated FPT for all trips over radii ranging from 100 to 5000 m in 25-m increments [53]. For each path, we calculated the variance of log-transformed first-passage time values (transformed to make variance independent of the mean) for each radius (Additional file 1: Figure S4A). The resulting peak in variances indicates the scale at which the organism is concentrating its activities; however, this scale may vary by individual. We found a common scale for analysis by selecting the radius with highest mean variance when averaged across all paths [53].

The mean variance of log FPT peaked at a radius of 250 m (Additional file 1: Figure S4A). This value was selected as the FPT radius for all tracks. A total of 99 locations could not have FPT values calculated because they occurred too close to the beginning or end of a given trip. To determine a threshold FPT value for separating foraging from commuting, we fit a bimodal Gaussian mixture distribution with the function “normalmixEM” from the R package “mixtools” to the distribution of ln(FPT). We used the 95% upper confidence interval between bimodal peaks of ln(FPT), which occurred at 142 s (Additional file 1: Figure S4B), to set the threshold between foraging and commuting FPT values.

### Hidden Markov model

A hidden Markov model (HMM) assumes an animal has more than one hidden behavioral state with characteristic speed and turn angles that can be modeled using stochastic processes [12]. We used the R package “momentuHMM” to fit HMMs to all tracks [54]. We used linear interpolation at 15 s increments to fill in missing GPS values caused by signal loss or device malfunction to address the HMM assumption of constant sampling rate. We used a two-state model to define behavioral states.

Initial step length, or distance between sampling locations, parameters for the two-state HMM were estimated from a mixed normal distribution of the step length of all individuals using the function “normalmixEM” (state 1: mean 70.2 m, SD 27.6 m; state 2: mean 160.8 m, SD 23.0 m). The HMM estimated gamma distributions for step length parameters (state 1: mean 46.7 m, SD 26.9 m; state 2: mean 83.6 m, SD 21.1 m) and von Mises distributions for turn angles (state 1: mean 0, concentration 0.23; state 2: mean 0, concentration 11.01). State 1 has a shorter step length and uniform turn angle distribution, while state 2 has longer step lengths and a turn angle concentrated around 0. Previously, these states have been termed area restricted search and exploratory movements, respectively [12, 55], and were assigned as foraging and commuting behaviors in this study. Transition probabilities from foraging to commuting and commuting to foraging were 3.9 and 7.2% respectively (Additional file 1: Table S3). A Jarque-Bera test of normality for step length (X^2^ = 301.59, df = 2, *p* < 0.001) and turn angle (X^2^ = 5.38, df = 2, *p* = 0.07) indicated that the distribution for step length deviated from normality (Additional file 1: Figure S5, Table S4). We also fit a three-state HMM, which had a slightly higher median but lower mean balanced accuracy (see Additional file 1: Figure S6, Table S5). To investigate the impact of sampling rate, we performed the same HMM analysis on subsampled data from 15 s to 10 min intervals by 15 s increments and found that median HMM performance gradually decreased with subsampling (linear regression: slope = − 5.2 × 10^− 5^, F(1,38) = 16.34, R^2^ = 0.3, *p* = 0.0002; Additional file 1: Figure S7).

### Expectation-maximization and binary clustering

Expectation-maximization and binary clustering (EMbC) is an unsupervised clustering algorithm that uses maximum likelihood estimation of a Gaussian mixture model [14]. EMbC is a parameter-free method that groups velocity and turn angle into low and high values, creating four clusters of intuitive biological interpretation: low velocity and low turn angle (LL - resting), low velocity and high turn angle (LH – intensive search or ARS), high velocity and low turn angle (HL – commute), and high velocity and high turn angle (HH – extensive search or possibly predator avoidance) [14]. We used the R package “EMbC” to annotate all tracks into these clusters (Additional file 1: Figure S8). We aggregated two search clusters, LH and HH, into foraging locations and grouped the two remaining clusters (LL and HL) into commute locations. We chose these groupings of the data because the HH category does not reach very high speeds, suggesting it is still ARS movement, and this particular grouping gave the highest performance (Additional file 1: Figure S9, Table S6).

### Change point analysis of correlated velocity movements

Correlated velocity movement (CVM) refers to a family of continuous-time movement models where velocities follow Ornstein-Uhlenbeck processes. We used the R package “smoove” (github.com/EliGurarie/smoove) to estimate CVM and conduct likelihood-based change point analyses [15, 16]. We used the random movement or unbiased CVM (UCVM) model to partition each trip into behaviorally consistent segments and estimate speed and autocorrelation for each segment. To estimate speed, autocorrelation, and potential change points, we used a 2 min window (8 location points), as increased window length would be less sensitive to detecting short foraging bouts and did not improve performance (Additional file 1: Figure S10). After each estimate, the window was shifted down the track 1.25 min (5 location points) and the first step was repeated. For one trip (Viv19 on 2015-06-02 UTC-7) a 2 min window length would not converge, and a window length of 2.5 min was used. The resulting peaks in log likelihood values were then used as candidate change points. Change points could not be less than 30 s apart, limiting the minimum duration of each segment. Change points were further thinned by recursively fitting CVM models to each segment with and without a final change point and then selecting the model with the lowest Bayesian Information Criterion (BIC) score.

Correlated velocity change point (CVCP) analysis determines behavioral states by refitting each segment to an advective CVM (ACVM) and choosing the model with the lower BIC score. UCVM uses a Gaussian distribution for position and velocity, with the long-term mean position equal to the initial position, whereas ACVM has a mean non-zero advective velocity. We labeled UCVM segments as foraging because they resembled ARS movement, and we labeled ACVM segments as commuting because they tend to be straight and fast. For each CVCP segment, we record the model fit as either unbiased or advective CVM, corresponding to foraging or commuting for all locations within that segment and parameter estimates of root mean squared speed and tau, a measure of time over which data are autocorrelated (Additional file 1: Table S7).

### Evaluation of segmentation methods

We assessed performance of each movement segmentation method using presence and absence of buzzes which were detected in the on-board audio recordings and assigned to the nearest GPS location. We expected that a good segmentation method would accurately predict the presence of buzzes during predicted foraging and absence of buzzes during predicted commuting. We scored buzz occurrences during foraging segments as true positives and absence of buzzes in commute segments as true negatives. True positive rate (TPR), or sensitivity, is the rate of choosing the correct value when the underlying condition is true. Here, TPR is the number of matches between buzzes and predicted foraging over the total number of buzzes. True negative rate (TNR), or specificity, measures the proportion of negatives that are correctly labeled.

Since buzzes are relatively rare in our recordings, we did not use accuracy, which assumes a balanced dataset of true positives and true negatives. For example, if a method determines that all locations are “foraging” or all locations are “commuting”, this would yield a 100% TPR or 100% TNR, respectively. Therefore, we used the average of TPR and TNR, referred to as “balanced accuracy”, which punishes methods for selecting too many events from the same class (i.e. foraging or commuting). A balanced accuracy of 50% corresponds with a random guess, while a perfect true positive and true negative rate would yield a balanced accuracy of 100%. Furthermore, balanced accuracy is more appropriate for unbalanced data because it weights TPR and TNR equally even if the number of observations in each is different [56].

After computing the balanced accuracy for each method per individual, we then tested whether there were significant differences in TPR, TNR and balanced accuracy between the methods using a Friedman’s test [57]. If there were statistically significant differences between methods, we then performed Wilcoxon paired-sample tests with Bonferroni correction to determine which models differ from the model with highest balanced accuracy.

We then compared the mean speed and turn angle associated with buzzes on each bat trip to mean speed and turn angle identified for foraging by each of the five segmentation methods, as explained below, using Wilcoxon paired-sample tests with a Bonferroni correction. Finally, we compared segmentation parameters (percentage of track foraging, mean foraging segment duration, and mean number of foraging segments) between the top performing method and all other methods, to determine if methods were segmenting trips similarly to the top performing method.

All analyses were conducted using R version 3.4.3 (R Core Team 2017).

## Results

### Trip summary

Trip duration and number of buzzes recorded varied among individuals. After removing locations within 100 m of each bat’s initial position on the island, there were 12,038 GPS locations, and 688 buzzes. The duration of trips was 3.4 ± 1.8 h (mean ± SD, range 0.9–6.4, *N* = 15), with total distance per trip of 42.6 ± 25.8 km (range 6.79–89.80 km, N = 15), and number of feeding buzz events per trip of 45.9 ± 32.8 (range 5–138, *N* = 15) (Additional file 1: Figure S1, Table S1). Given the 10% audio duty cycle, the actual number of feeding buzzes emitted is about ten-times greater (as we confirmed by tagging bats with 50–100% duty-cycle tags, Additional file 1: Figure S2). The distribution of times between buzzes has a long right-tail towards higher time intervals, which has an exponentially decreasing shape, indicating that most buzzes occur in clusters, i.e., in foraging bouts (Additional file 1: Figure S11).

### Model performance

The performance of behavioral classification methods was assessed for each bat flight using the true positive and true negative rates (Additional file 1: Table S8). Some methods over-predicted commuting behavior (e.g. EMbC and kmC), suggesting that they failed to detect or fully capture many foraging bouts. While kmC had the highest median TNR, followed by EMbC, HMM had the highest TPR and was moderately higher than FPT and CVCP (Fig. 4a). Balanced accuracy showed significant differences among methods (Friedman χ^2^ = 20.69, df = 4, *p* < 0.001). HMM had the highest median balanced accuracy (67.3%), followed by CVCP (64.1%), EMbC (63.2%), kmC (62.7%), and FPT (61.5%), though post-hoc tests indicated that FPT and CVCP did not significantly differ from HMM (Wilcoxon paired test with Bonferroni correction: FPT vs. HMM *p* = 1; CVCP vs. HMM, *p* = 0.15; Fig. 4b). Variation in balanced accuracy within methods could not be explained by distance traveled (ANCOVA: F_4,65_ = 0.37, *p* = 0.83) or number of buzzes during a trip (ANCOVA: F_4,65_ = 0.38, *p* = 0.82).

### Movement parameters associated with buzz locations

All five segmentation methods identified two states: foraging with low speed and high turn angle and commuting (Table 2). Foraging speeds and turn angles at locations where buzzes were detected agreed with most segmentation estimates of foraging speed and turn angle; however, speed associated with buzzes (mean ± SD: 3.4 ± 0.5 m/s) differed from foraging speed estimated by kmC (Wilcoxon paired test with Bonferroni correction: *p* = 0.004) and turn angle associated with buzzes (mean ± SD: 72.5 ± 16.7°, N = 15) differed from turn angles estimated for foraging by kmC and EMbC (*p* < 0.001 and *p* < 0.001 respectively; Table 2).

**Table 2:** Mean and standard deviation of flight parameters for behavioral states identified by each segmentation method

	Foraging		Commuting		
Method	Speed (m/s)	Turn angle |degrees|	Speed (m/s)	Turn angle |degrees|	Omitted points
kmC	2.87 ± 0.20**	130.3 ± 3.1***	5.05 ± 0.40	19.2 ± 3.2	30
FPT	3.48 ± 0.46	69.0 ± 11.2	5.49 ± 0.61	21.5 ± 13.0	99
HMM	3.13 ± 0.22	83.4 ± 8.1	5.65 ± 0.42	13.9 ± 1.5	0
EMbC	3.12 ± 0.22	112.2 ± 5.9***	5.13 ± 0.39	17.3 ± 2.4	0
CVCP	3.48 ± 0.50	74.8 ± 12.2	5.33 ± 0.34	21.8 ± 7.2	0
Buzz	3.36 ± 0.54	72.5 ± 16.7	NA	NA	NA

Wilcoxon pairwise comparisons between buzz occurrence and foraging parameters for each method with Bonferroni correction (see text)

**p-value < 0.01, ***p-value < 0.001

### Predicted foraging events

**Fig. 3:**
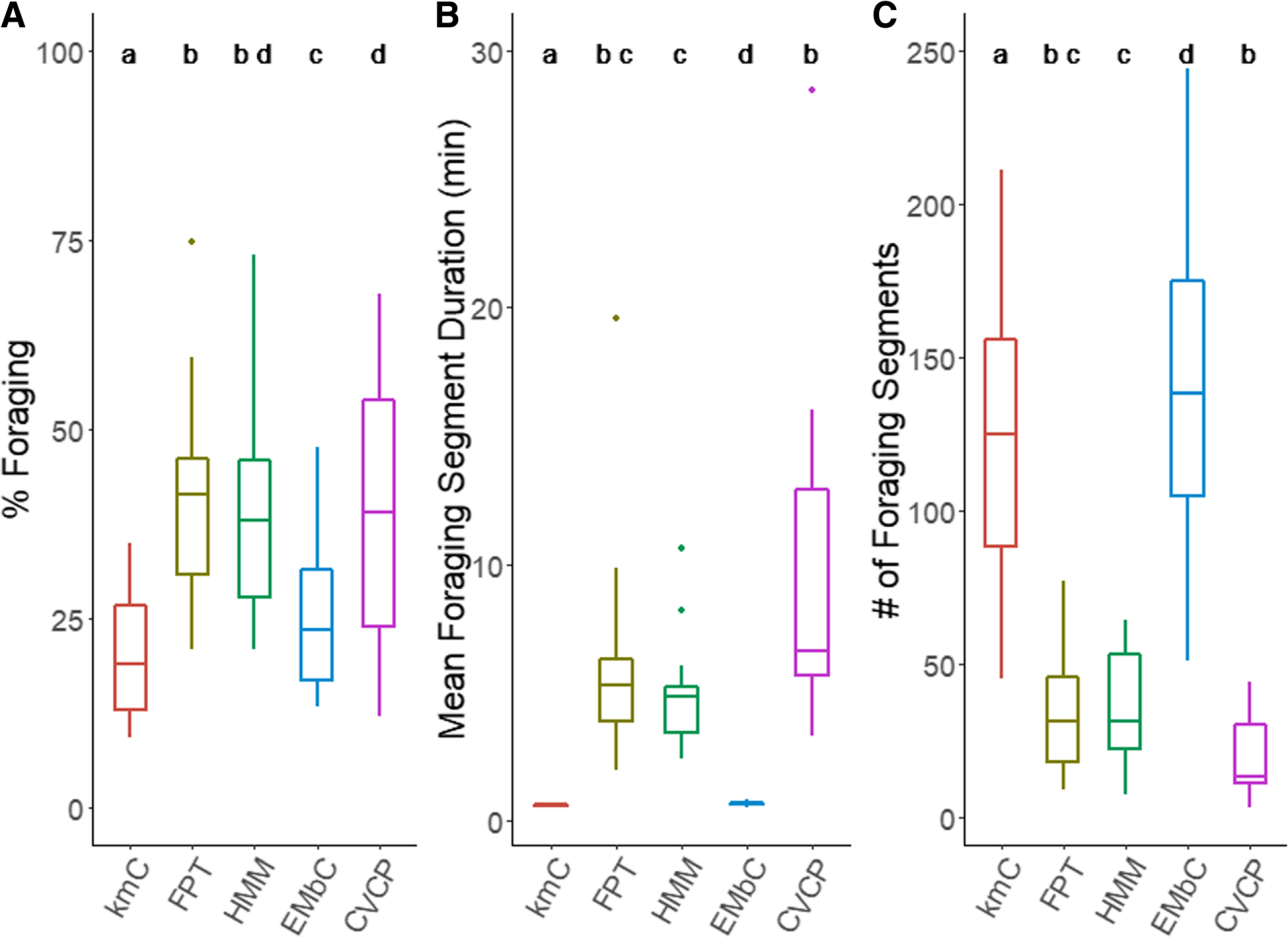
Box plots showing (**a**) the percentage of a trip in foraging behavior, (**b**) the number of locations in a foraging segment, and (**c**) the number of segments for each segmentation method (*N* = 15). Different letters above boxplots represent significant differences in paired Wilcoxon sign rank tests after Bonferroni correction

**Fig. 4:**
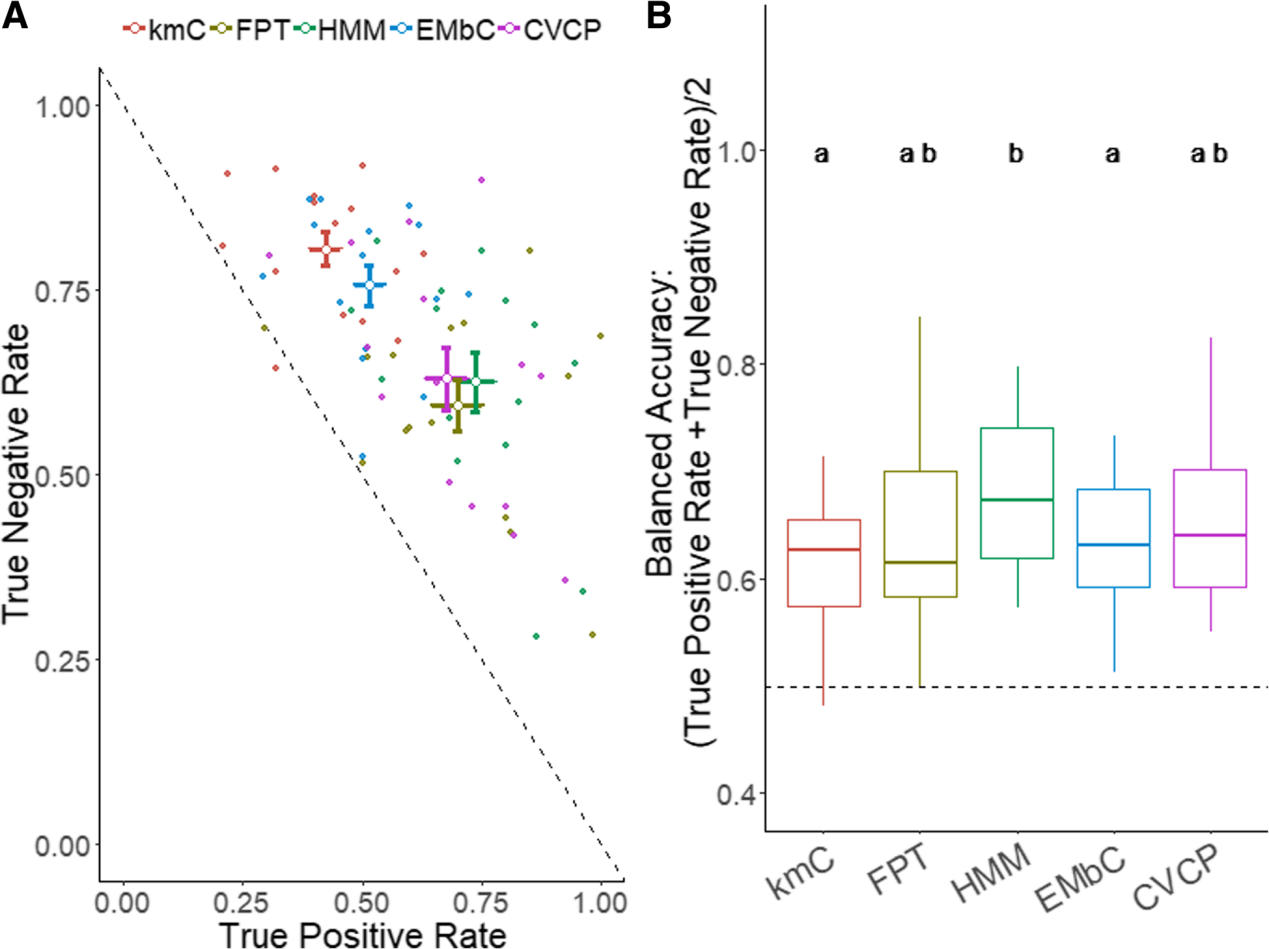
**a** Scatter plot of true positive rate against true negative rate (each point represents a bat flight and method combination) and (**b**) box plot of the balanced accuracy for each trip (N = 15). The scatter plot includes mean values of each method with standard error for true positive rate and true negative rate (**a**). A dashed line is included in both plots to show 50% balanced accuracy and values above the line represent good classification. Different letters above boxplots represent significant differences in paired Wilcoxon sign rank tests after Bonferroni correction (**b**)

The segmentation methods also varied in how much foraging each predicted for each trip (Fig. 3). Percent of trip foraging, mean foraging segment duration, and number of foraging segments showed significant differences among methods (Friedman’s test for percent foraging: χ^2^ = 47.41, df = 4, *p* < 0.001; segment duration: χ^2^ = 53.17, df = 4, *p* < 0.001; number of segments: χ^2^ = 54.03, df = 4, *p* < 0.001). Post-hoc tests indicated that proportion of time spent foraging in a trip predicted by FPT and CVCP did not significantly differ from that predicted by HMM (Wilcoxon paired test with Bonferroni correction: FPT vs. HMM *p* = 0.8; CVCP vs. HMM, *p* = 1; Fig. 4b). FPT, HMM, and CVCP identified foraging during about 40% of each trip; whereas, EMbC predicted about 25% and kmC predicted about 20% (Fig. 3a). The duration and number of segments, i.e. changes in behavioral state between foraging and commuting, per trip differed substantially between most methods (Fig. 3b, c) with the exception of FPT and HMM which predicted very similar foraging segment duration (4–6 min) and number (35–40).

## Discussion

We present the first evaluation of segmentation methodology performance in a free flying bat. Individual foraging trips varied considerably in duration and number of buzzes (Additional file 1: Table S1), providing a complex set of data for each segmentation method. Our goal was to identify the best performing segmentation method for fish-eating bat foraging trips. We found that 1) HMM had the highest median balanced accuracy, 2) HMM, FPT, and CVCP foraging segments predicted speed and turn angles similar to those for buzz locations and 3) HMM segmentation was most similar to FPT in terms of percent of trip foraging, duration of segments, and number of segments in a trip. These results point to HMM as the best segmentation methodology with FPT as a useful alternative. Overall, balanced accuracy was limited (no greater than 67% overall, and 84% for the best-identified individual trip) because buzzes are rare events during a foraging bout, so segmentation methods will inevitably identify more locations as foraging than will buzzes. Variation in accuracy of segmentation methodology may also be influenced by sampling design, which can be controlled by researchers, or by nuances of animal behavior, which can lead to biological insights.

### Predicting foraging behavior

Fish-eating bat trips typically consist of an outward commute from the roost, composed of straight fast flight, followed by several foraging bouts, which can either have short transits or longer commutes connecting them, and finally a commute back to the roost [26]. All methods identified commuting phases with higher speeds and lower turn angles than during foraging bouts, consistent with animals searching for unpredictable patchy resources [26, 37, 58]. While generally robust at identifying long commute or foraging movements, segmentation methods often struggle with how finely they parse short foraging bouts and transits.

**Fig. 5:**
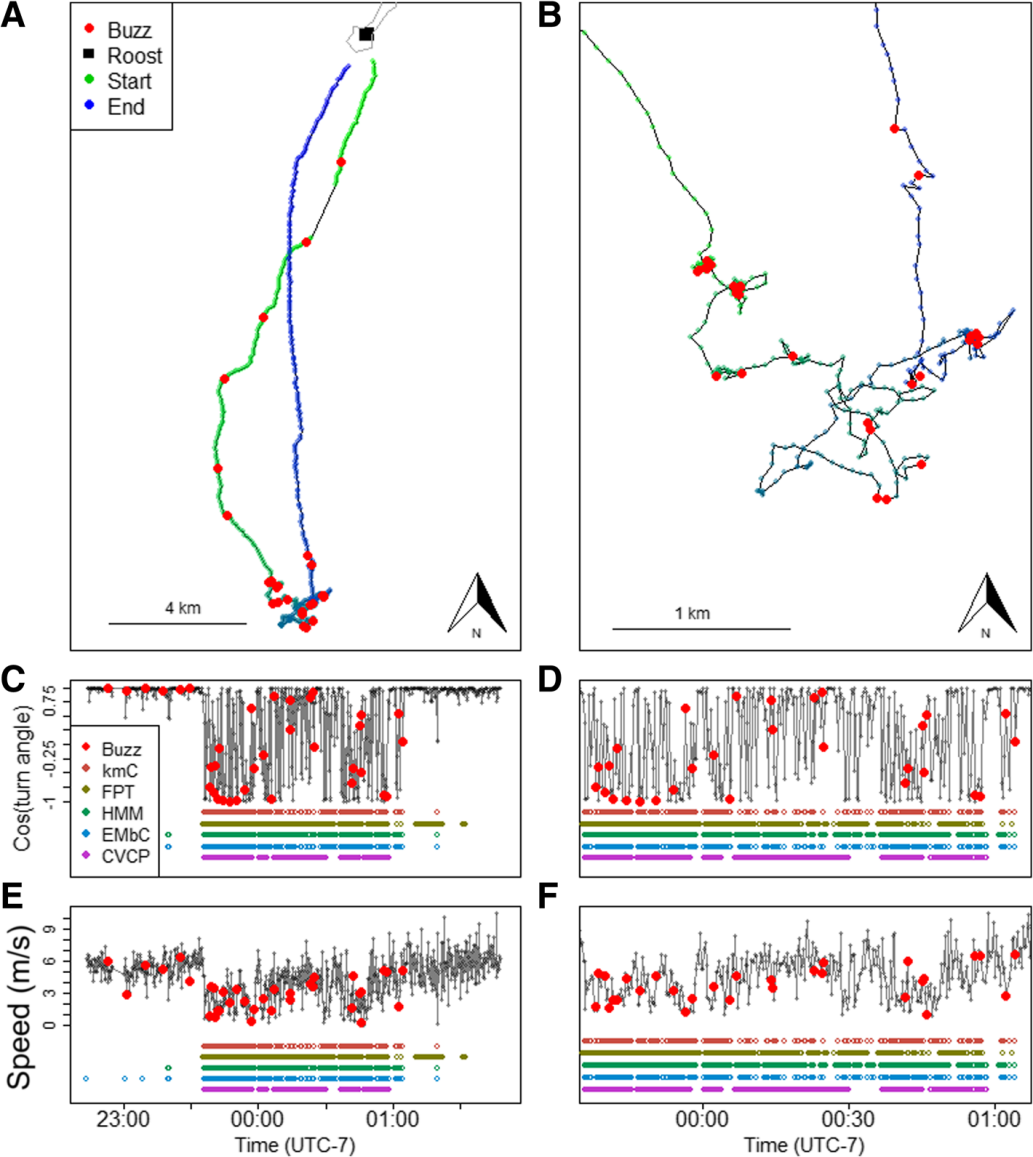
Example trip showing (**a**) the flight trajectory, (**c**) cosine of the turn angle for the entire flight, and (**d**) speed for the entire flight. (**b**) displays a close-up of the foraging area in (**a**), (**d**) cosine of the turning angle for (**b**), and (**f**) speed for (**b**). Buzzes are overlaid as red circles in all plots and segmentation methodology predictions of foraging are shown in different colors below speed and turn angle plots (**c**-**f**). The number of foraging segments identified in this trip varies between methods (kmC: 145; FPT: 80; HMM: 31; EMbC: 138; CVCP: 13)

Inspection of an example trip provides insight into how each method performs (Fig. 5). All methods converged on a large foraging area at the furthest point from the roost and, after lining up foraging segments in time (Fig. 5), most methods agree on the beginning and end of some segments. However, coverage and change points for each method vary. All methods show several breaks in the foraging segment, increasing from CVCP, which only has a few foraging segments separated by a few breaks, to HMM and FPT, which have more breaks, to kmC and EMbC, which break the foraging event into over 100 brief events. Methods with high numbers of segments, such as EMbC and kmC, consistently had lower true positive rates, suggesting that finely parsing foraging results in more missed buzzes. Yet, some of these breaks are real events in which the bat transits between small patches, such as at 2:00 (UTC-7). Other breaks in foraging segments could reflect an ARS that follows a drifting resource patch and therefore has an increased speed and more uniform direction. These identifications would then be considered “false negatives” of foraging detection and are more likely to occur with the unsupervised methods, EMbC and kmC.

### Evaluating foraging with buzzes

Buzzes appear to be concentrated in foraging bouts, supporting the hypothesis that prey capture attempts increase during foraging movements [10]. However, some buzzes were clearly recorded along a straight outgoing track, which all methods classify as commuting (Fig. 5). There was no obvious change in speed or turn angle during these events, and they were consequently missed by all the classification algorithms, increasing the number of false negatives. It is possible that these buzzes occur during brief foraging bouts in which the bat attempted to attack prey on the water surface while commuting. Another possibility is that these brief events occur when fish-eating bats opportunistically encounter aerial prey, a behavior occasionally observed [36]. The fact that buzzes are not exclusively limited to ARS suggests some plasticity in the foraging behavior of *M. vivesi*.

Balanced accuracy values are also likely reduced because audio was not sampled continuously, possibly leading to missed buzzes in foraging segments. Analysis of all-night continuous audio recordings revealed that a 10% duty cycle (0.5 s every 5 s) captured 77% of foraging bouts (Additional file 1: Figure S2), demonstrating that we captured most, but not all, prey capture attempts. Unfortunately, those continuous audio recordings did not include GPS sampling and therefore cannot be used to determine how each segmentation method would perform against a more complete audio data set. Future work should aim to collect audio at a higher duty cycle in tandem with additional independent behavioral monitoring devices, such as an accelerometer, which may detect changes in wingbeat patterns, or barometric pressure, which would reveal when bats are close enough to the surface of the ocean to capture prey.

### Evaluation of methods

HMM, while not significantly better than all methods, had the highest median balanced accuracy and predicted movement metrics similar to those measured at buzz locations. Where they have been independently validated with behavioral signals, HMM’s have shown promise in accurately assigning behavioral states across a variety of taxa, e.g. elephant seals [59] and gannets [10]. HMM’s define both the state distribution (the distribution of input turn angle and step length) and the transition probability between states (Additional file 1: Table S3). In principle, HMM’s require data recorded at regular sampling intervals with negligible measurement error and can be influenced by autocorrelation in the data since there is an assumption of serial independence among turning angles and step lengths. Diagnostic plots of the HMM pseudo-residuals indicate a lack of fit because the independence of the data are often violated. Nonetheless, in our case HMM still outperformed other methods, suggesting that, in practice, model misspecification is not a fatal flaw. Timescale of autocorrelation estimates from CVCP in foraging tended to be lower than 15 s, indicating that foraging movements were independent, possibly explaining the performance of HMM in identifying foraging. Furthermore, we investigated how subsampling to coarser sampling resolutions influenced performance and found a significant but subtle decrease in median balanced accuracy, suggesting that HMM performance is generally robust to GPS sampling rate (Additional file 1: Figure S7). We also fit a three-state HMM. Despite a lower AIC and slightly higher median balanced accuracy of a three-state HMM, a two-state HMM had a higher mean balanced accuracy and less variation than the three-state model (Additional file 1: Figure S6, Table S5).

Due to limited recordings per individual, all methods assumed no individual variation. Nightly differences in oceanographic conditions, weather, prey distribution, and social context could influence the way an animal commutes or forages and consequently affect method performance, especially if trips are aggregated for analysis [10, 25]. We limited our analyses to just movement and buzzes to simplify method comparison; however, many segmentation analyses are performed for habitat selection analysis or use environmental parameters with movement to identify behavior state changes [11, 60, 61]. While behavioral states annotation from all five methods can be used for habitat selection, HMM can include environmental covariates in the model and clearly present how those covariates influence transition probabilities, which is an additional advantage of this method. Alternatively, the transitions and parameters obtained from other methods can be analyzed with respect to environmental covariates post hoc. For example, the probability that a foraging phase might have an advective term from the CVCP could be modeled with respect to winds or currents to indirectly explore hypotheses related to drifting prey patches. As another example the frequency of extremely short foraging bouts from the clustering algorithms might be related to environmental conditions associated with less concentrated and patchier prey fish aggregations.

One of the earliest and heuristically simplest of the segmentation methods, FPT is still one of the most commonly used methods to identify foraging areas [10, 53, 60], and – because it is explicitly spatial rather than velocity-based – can outperform more statistically complex tools [8]. In our study, FPT had the most variable accuracy among individual trips. FPT was most similar to HMM in performance, despite having lower median balanced accuracy, and some of the individual trips had extremely high accuracy (up to 84%). We did not explore the effect of different radii and thresholds on balanced accuracy but instead chose a radius that maximized the overall variation in FPT and a cut-off between foraging and commuting that was at the upper confidence limit for the first peak in the ln(FPT) histogram. The fact that only one radius and threshold were used likely explains much of the variability in accuracy, since both the radius and threshold may change according to individual or environmental conditions. For example, bats may be influenced by drifting prey or strong winds, which may compromise the ARS pattern and require a larger radius or threshold to separate behaviors. Replicates of trips for the same individual across environmental conditions, as well as measurements of the relevant abiotic conditions, could be combined with an FPT analysis to gain further insights into the context of fish-eating bat behavioral states.

CVCP requires a large enough analysis window to reliably estimate movement parameters and change points. By design, CVCP segments are more likely to correspond to behavioral switches than spurious changes in movement. Though less sensitive than other methods, its balanced accuracy was comparable to HMM and FPT. Furthermore, CVCP is the only method that provides a completely parameterized continuous-time movement model that is defined in terms of biologically meaningful parameters (e.g. advective and random mean speeds and characteristic time scales of auto-correlation, [16]), which makes it possible to compare the mechanistic features of larger-scaled commuting and foraging behaviors across individuals or foraging trips. Estimates of tau, which is an estimate of the time scale at which the velocity of an animal’s movement is autocorrelated, are typically higher than the 15 s GPS sampling schedule in commuting and less than 15 s for foraging locations (Additional file 1: Table S7). This suggests that the 15 s sampling rate is sufficient for characterizing the commuting movements, but that the movements (and decisions) that the bat is performing while foraging occur at a faster rate, requiring an even higher location sampling rate or ancillary information (e.g. from an accelerometer) to explore the foraging behaviors in higher detail. This result does, however, suggest that the independence assumption behind the HMM is essentially satisfied, at least for the foraging state. It is further worth noting that the CVCP is perhaps best suited to distinguish between highly localized foraging and foraging for a drifting patch, since it can fit a model with advection that still has varying degrees of tortuosity and movement speeds.

The two unsupervised methods, EMbC and kmC, produced similar segmentation patterns of bat foraging trips. Pattern description methods, such as kmC, do not require a predefined length for segments and can therefore detect very brief foraging bouts (Fig. 3b). It is likely that EMbC is attempting to overfit the movement data and defines states that do not occur during these trips, such as resting. However, by aggregating states, we demonstrate that this method can still be useful on data that has been filtered to exclude time in the roost. While these methods identified most foraging sites, they provided unreliable estimates of parameters associated with foraging due to their lower performance.

## Conclusion

Despite variation in movement statistics, performance, as measured by balanced accuracy, was not very different among methods. While performance is highest with HMM, technical constraints might lead some researchers to use simpler or faster methods, like FPT, that do not require parameter estimation, though recently developed R packages like “momentuhmm” have made the fitting of HMM models to movement data much more accessible [54]. Our results do indicate that the choice of segmentation method can lead to dramatically different movement statistic estimates, such as number of foraging bouts, percentage of time spent foraging, and locations of foraging areas. It is therefore important to be aware of the assumptions and limitations of each algorithm, as well as each tool’s sensitivity to sampling rate, missing locations, localization accuracy [59, 62] and individual differences [13]. Ultimately, the research question should inform method selection. Evaluating biophysical parameters such as speed and time scales of movement is easier with a more realistic movement model [16], while identifying covariates that influence the rate of behavior switching between stereotyped behaviors will require a state space model. In many cases, important insights can be made through “triangulation” – i.e. by using several tools and comparing the outputs. As animal movement data become more readily available, it will be increasingly possible to validate behavioral annotation methods. In species that lack sufficient observational data to calibrate behavioral state estimates, such as animals with cryptic foraging behaviors, researchers must decide whether their assumptions about behavior reflect reality.

## Additional file

Additional file 1: Supplemental figures and tables. (DOCX 270 kb)

## Data Availability

Hurme E, Gurarie E, Greif S, Herrera M LG, Flores-Martínez JJ, Wilkinson GS, Yovel Y (2019) Data from: Acoustic evaluation of behavioral states predicted from GPS tracking: a case study of a marine fishing bat. Movebank Data Repository. 10.5441/001/1.kk3bg2f4

## Abbreviations

AIC: Akaike Information Criterion
ARS: Area-restricted search
BIC: Bayesian Information Criterion
CVCP: Correlated velocity change point analysis
CVM: Correlated velocity movement
EMbC: Expectation maximization and binary clustering
FPT: First passage time
g: Grams
GB: Gigabyte
h: Hour
HMM: Hidden Markov models
IPI: Inter-pulse interval
kmC: k-means clustering
m: Meters
min: Minutes
s: Second
SE: Standard error
TNR: True negative rate
TPR: True positive rate
