# Supplementary material for: Acoustic evaluation of behavioral states predicted from GPS tracking: a case study of a marine fishing bat
Source: Mov Ecol. 2019 Jun 14;7:21. doi: 10.1186/s40462-019-0163-7 (PMC6567457; doi:10.1186/s40462-019-0163-7)
Supplement: Supplementary file 1 — Supplemental figures and tables. (DOCX 270 kb) [file 40462_2019_163_MOESM1_ESM.docx]

**Additional file 1: Figures and Tables**

Acoustic validation of behavioral states predicted from GPS tracking: a case study in a marine foraging bat

Edward Hurme, Eliezer Gurarie, Stefan Greif, L. Gerardo Herrera M., José Juan Flores-Martínez, Gerald Wilkinson, Yossi Yovel


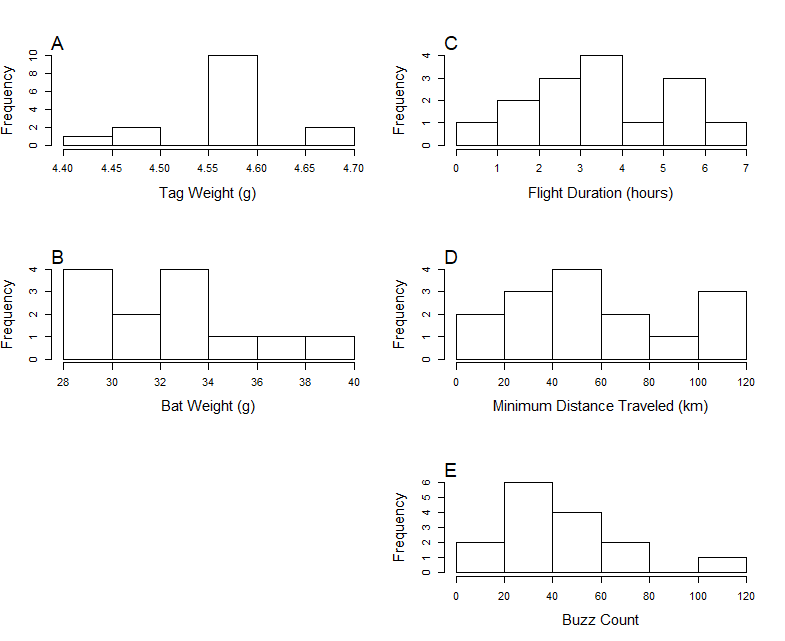
**Figure S1:** Histograms of the flight parameters of foraging bats.


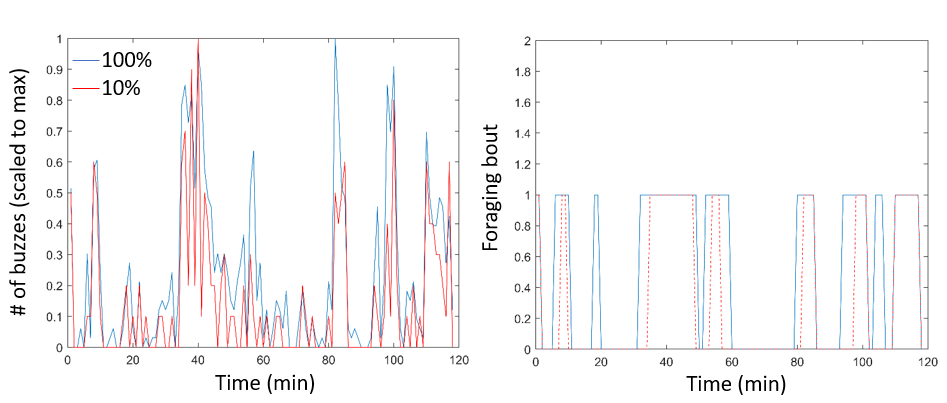


**Figure S2: Validation of audio sub-sampling. (A)** Normalized buzzes per minute are presented for the 100% duty cycle (blue) and the 10% under-sampled (red) recordings. Both graphs were normalized relative to their maximum for comparison. The very strong correlation between the two graphs demonstrates how sub sampling still allows foraging bout detection. **(B)** Foraging bouts detected according to the buzzes (as a function of time) for the 100% duty cycle (blue) and the 10% under-sampled (red) data. Foraging bouts are depicted by values of ‘1’. Foraging bouts were defined as minutes in which the number of buzzes was above 20% relative to the max (bouts of 1 minute were removed and gaps of 1 minute were connected). This definition, relative to the max, allows a comparison between sampling strategies and as can be seen, 7 of the 9 bouts are detected in the under sampled data. Both A and B represent data of a single bat. In total, we repeated this analysis for three bats (see results in main text).


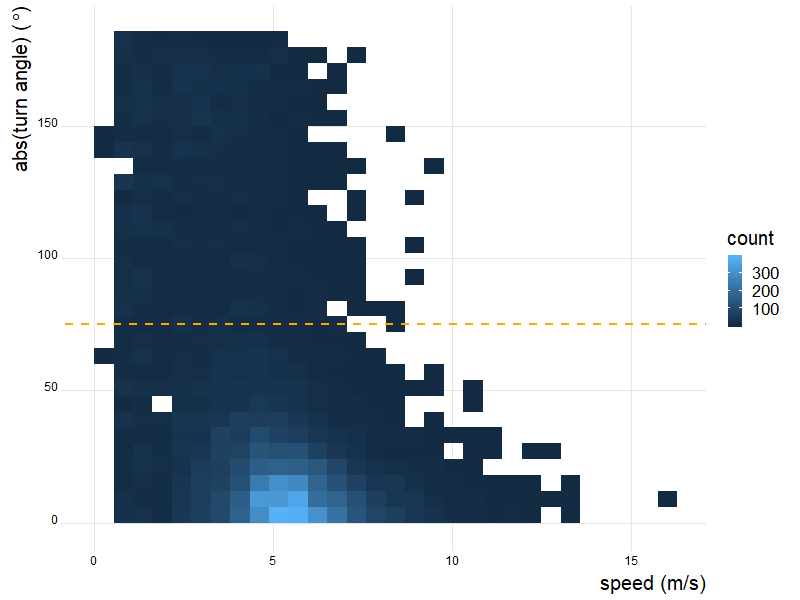
**Figure S3:** Distribution of speed and absolute turn angle values for all GPS locations. An orange dashed line indicates the boundary between k-mean states, with foraging occurring above 75º and commuting below 75º.


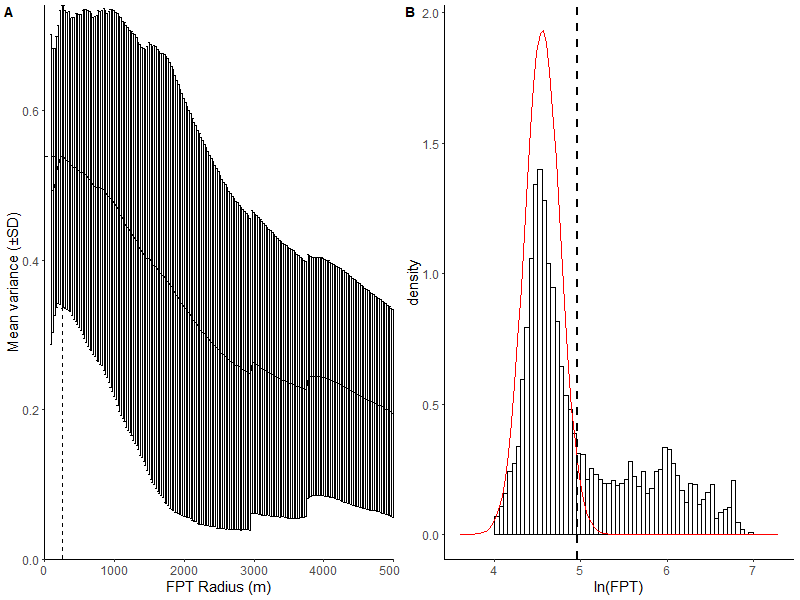
**Figure S4:** (A) Whisker plots of mean variance of first-passage time values over a range of radii from 100 m to 5000 m. A dashed line is overlaid to represent the highest mean value, which occurs at a radius of 250 m. (B) Histogram of the natural log of first-passage time values calculated for all bats at a radius of 250 m. A threshold (dashed line) between high (commuting) and low (foraging) FPT values is selected at the upper 95% confidence interval of a gaussian curve fit to the lower peak at 142 s.


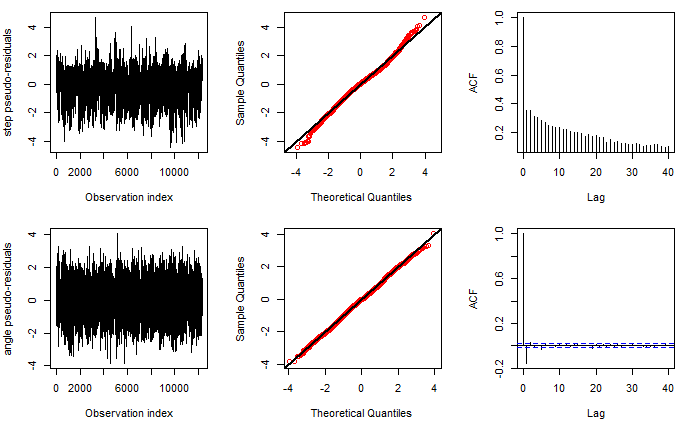
**Figure S5:** Psuedo-residual, QQ, and autocorrelation function plots of step length and turn angle modeled by a two state HMM.


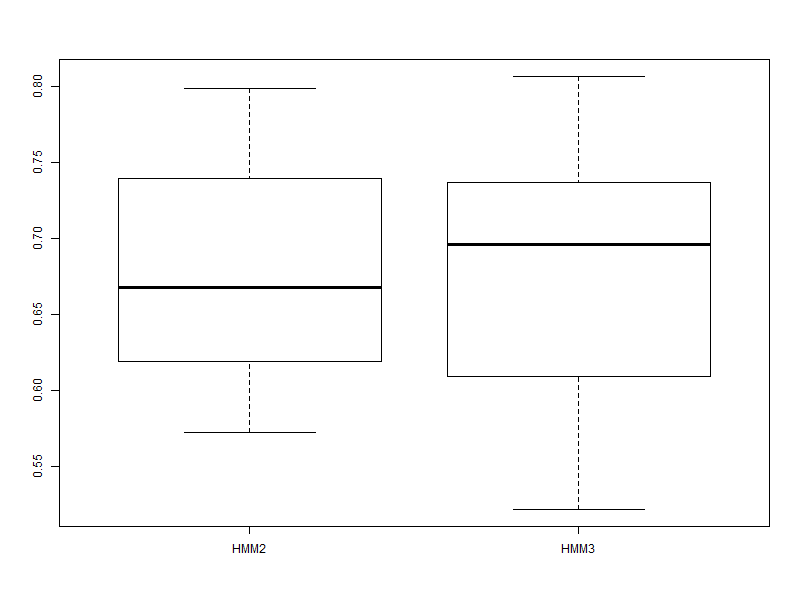


**Figure S6:** Boxplot of the balanced accuracy for each bat flight (N = 15) for a two-state and three-state HMM.


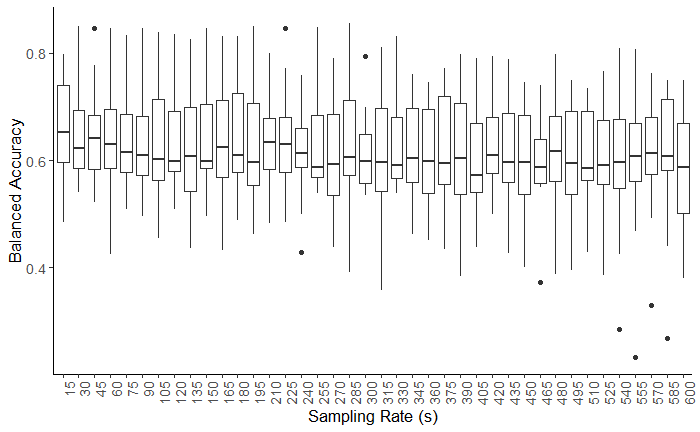
**Figure S7:** Balanced accuracy of hidden Markov models run on all tracks subsampled at different sampling rates.


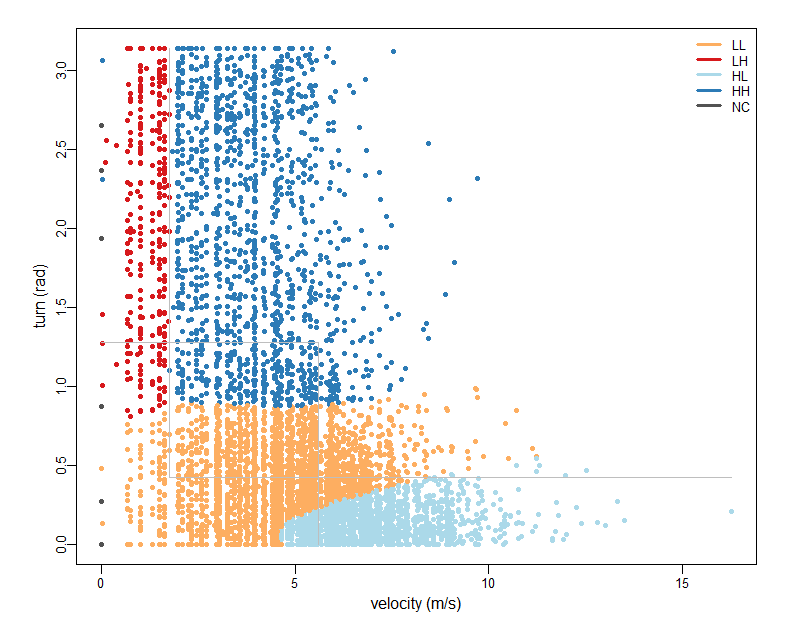
**Figure S8:** Expectation-maximization and binary clustering output. Colors correspond to the four categories of movement states defined by different clustering of the speed and turn angle.

**
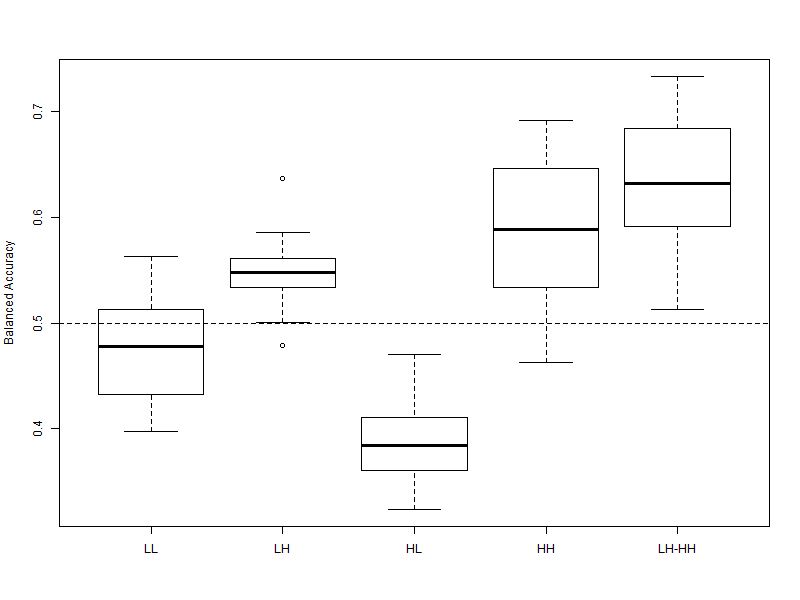
**

**Figure S9:** Boxplot of the balanced accuracy of each flight path (N = 15) among all EMbC states and a combination of the two highest performing states, low speed-high turn angle and high speed-high turn angle. This combination of states performed highest and was used as the foraging state for EMbC in the methodology comparison.


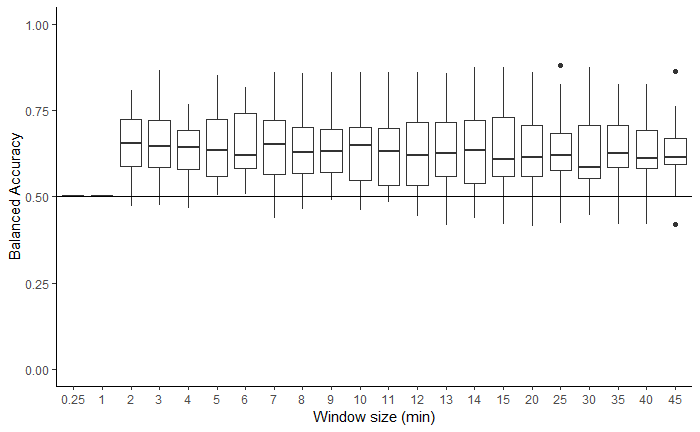


**Figure S10:** Boxplots of balanced accuracy of each bat flight (N = 15) for CVCP across a range of window sizes.

**
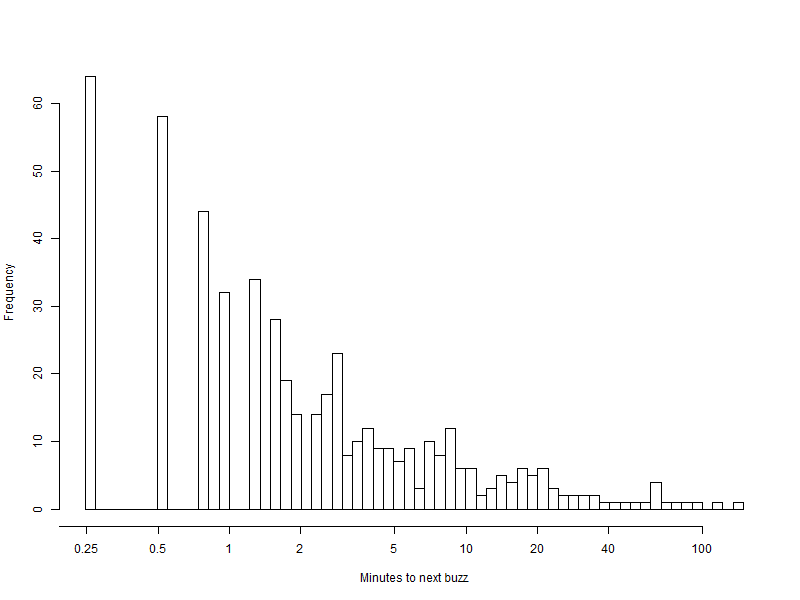
**

**Figure S11:** Histogram of the time in minutes to the next location with a buzz recorded.

**Table S1:** Summary of flight parameters of foraging bats.

| ID | Capture weight (g) | Tag weight (g) | Flight | Departure Time  (UTC-7) | Return Time  (UTC-7) | Minimum Distance  Traveled (km) | Duration (h) | Buzz Count | Audio files used (not filtered) | GPS fixes (not filtered) |
| --- | --- | --- | --- | --- | --- | --- | --- | --- | --- | --- |
| 5 | 29.5 | 4.4 | 1 | 5/27/2015 22:40 | 5/28/2015 1:58 | 34.28 | 3.3 | 78 | 2304 | 768 |
| 6 | 33.8 | 4.5 | 1 | 5/27/2015 22:16 | 5/28/2015 2:29 | 45.4 | 4.2 | 58 | 2967 | 989 |
| 6 | NA | 4.5 | 2 | 5/28/2015 21:54 | 5/29/2015 0:56 | 37.26 | 3.03 | 69 | 2112 | 704 |
| 8 | 33.8 | 4.6 | 1 | 5/29/2015 23:43 | 5/30/2015 2:47 | 30.5 | 3.07 | 35 | 2088 | 696 |
| 8 | 31.8 | 4.6 | 2 | 5/31/2015 21:08 | 6/1/2015 2:59 | 84.96 | 5.85 | 22 | 4134 | 1378 |
| 12 | 29.4 | 4.7 | 1 | 6/1/2015 20:48 | 6/1/2015 22:22 | 15.31 | 1.58 | 5 | 1140 | 380 |
| 12 | 29.4 | 4.7 | 2 | 6/2/2015 20:29 | 6/3/2015 1:34 | 51.19 | 5.07 | 49 | 3588 | 1196 |
| 14 | 36.1 | 4.6 | 1 | 5/30/2015 22:35 | 5/31/2015 2:29 | 54.25 | 3.9 | 25 | 2745 | 915 |
| 14 | NA | 4.6 | 2 | 5/31/2015 20:47 | 5/31/2015 21:38 | 6.79 | 0.85 | 10 | 615 | 205 |
| 15 | 33.3 | 4.6 | 1 | 5/31/2015 23:24 | 6/1/2015 2:06 | 46.77 | 2.7 | 34 | 1875 | 625 |
| 16 | 38.3 | 4.6 | 1 | 5/31/2015 21:02 | 6/1/2015 3:25 | 89.8 | 6.38 | 39 | 4509 | 1503 |
| 18 | 32.8 | 4.6 | 1 | 6/3/2015 20:25 | 6/4/2015 2:16 | 78.4 | 5.84 | 138 | 4134 | 1378 |
| 19 | 29.7 | 4.6 | 1 | 6/2/2015 20:57 | 6/2/2015 22:43 | 18.44 | 1.77 | 57 | 1278 | 426 |
| 21 | 34.2 | 4.6 | 1 | 6/2/2015 21:06 | 6/2/2015 22:11 | 15.2 | 1.1 | 24 | 792 | 264 |
| 24 | 30.1 | 4.6 | 1 | 6/5/2015 21:25 | 6/6/2015 0:02 | 29.82 | 2.63 | 45 | 1833 | 611 |
|  |  |  |  |  |  |  |  |  |  |  |

**Table S2:** Summary of flight parameters of acoustically monitored foraging bats tagged with vesper tags.

| ID | Capture weight (g) | Tag weight (g) | % body mass | Departure Time  (UTC-7) | | Return Time  (UTC-7) | | Duration (h) | Buzz Count | Recording Schedule |
| --- | --- | --- | --- | --- | --- | --- | --- | --- | --- | --- |
| Mviv17_16 | 28.2 | 4.1 | 14.5 | 6/9/2017 21:20 | 6/10/2017 0:14 | | 2.9 | | 135 | 26 s/ 26 s |
| Mviv17_49 | 30.5 | 4.3 | 14.1 | 6/22/2017 21:19 | 6/23/2017 1:43 | | 4.4 | | 267 | 7.5 s/ 15 s |
| Mviv17_60 | 32.2 | 4.3 | 13.4 | 6/26/2017 20:47 | 6/27/2017 0:15 | | 3.47 | | 1624 | 35 s/ 35 s |

**Table S3:** HMM state transition probabilities provide the overall probability of transitioning from one state to another or remaining in the current state.

|  | **State 1 – forage** | **State 2 – commute** |
| --- | --- | --- |
| **State 1 – forage** | 93.1% | 6.9% |
| **State 2 – commute** | 4% | 96% |

**Table S4:** Jarque Bera test of a three-state model

|  | **Step** | **Turn** |
| --- | --- | --- |
| **Χ^2^** | 203.14 | 9.85 |
| **df** | 2 | 2 |
| **p-value** | >0.001 | 0.007 |

**Table S5:** Comparison of 2- and 3-state HMM models.

| **HMM** | **State** | **Buzzes** | **Locations** | **TPR** | **Balanced Accuracy** | **AIC** | **dAIC** |
| --- | --- | --- | --- | --- | --- | --- | --- |
| 2-state | 1 | 450 | 4280 | 10.5% | Mean: 68.0% | 133971 | 3398 |
|  | 2 | 173 | 8043 | 2.2% | Median: 66.8% |  |  |
| 3-state | 1 | 386 | 3380 | 11.4% | Mean: 67.8% | 130573 |  |
|  | 2 | 201 | 4426 | 4.5% | Median: 69.6% |  |  |
|  | 3 | 36 | 4567 | 0.7% |  |  |  |

**Table S6:** EMbC states and a count of locations that correspond with buzzes and those that do not.

| **State** | **Speed** | **Turn** | **Locations with buzz** | **Locations without buzz** |
| --- | --- | --- | --- | --- |
| 1 | Low | Low | 247 | 5321 |
| 2 | Low | High | 91 | 592 |
| 3 | High | Low | 46 | 3701 |
| 4 | High | High | 216 | 1817 |
| 5 | NA | NA | 0 | 7 |

**Table S7:** Median and interquartile range of CVM parameter estimates.

|  | **Root mean squared speed (m/s)** | **Tau (s)** |
| --- | --- | --- |
| **UCVM (Foraging)** | 3.5, 2.7 – 4.9 | 17, 1 – 45 |
| **ACVM (Commuting)** | 5.3, 4.7 – 6.1 | 185, 91 – 308 |

**Table S8:** Comparison of foraging and buzz identification across all segmentation methodologies**.**

| **ID** | **Flight** | **Segmentation methods** | | | | | | | | | |
| --- | --- | --- | --- | --- | --- | --- | --- | --- | --- | --- | --- |
|  |  | **kmC** | | **FPT** | | **HMM** | | **EMbC** | | **CVCP** | |
|  |  | Foraging locations/  Total locations | Buzz hit/ total buzz | Foraging locations/  Total locations | Buzz hit/ total buzz | Foraging locations/  Total locations | Buzz hit/ total buzz | Foraging locations/  Total locations | Buzz hit/ total buzz | Foraging locations/  Total locations | Buzz hit/ total buzz |
| 5 | 1 | 141/766 | 36/72 | 316/762 | 73/72 | 269/768 | 68/72 | 178/768 | 40/72 | 319/768 | 68/72 |
| 6 | 1 | 221/987 | 37/54 | 292/979 | 17/50 | 377/989 | 55/54 | 278/989 | 43/54 | 375/989 | 47/54 |
| 6 | 2 | 210/702 | 31/63 | 414/696 | 56/63 | 353/704 | 47/63 | 243/704 | 34/63 | 394/704 | 51/63 |
| 8 | 1 | 167/694 | 20/35 | 306/693 | 21/35 | 295/696 | 29/35 | 197/696 | 23/35 | 271/696 | 23/35 |
| 8 | 2 | 200/1376 | 11/21 | 409/1371 | 16/21 | 355/1378 | 15/21 | 235/1378 | 14/21 | 262/1378 | 11/21 |
| 12 | 1 | 50/378 | 2/5 | 116/374 | 43590 | 103/380 | 43560 | 63/380 | 2/5 | 62/380 | 3/5 |
| 12 | 2 | 226/1194 | 10/48 | 518/1189 | 32/48 | 453/1196 | 26/48 | 280/1196 | 14/48 | 479/1196 | 26/48 |
| 14 | 1 | 87/913 | 5/23 | 309/908 | 14/23 | 259/915 | 43822 | 123/915 | 43731 | 189/915 | 9/23 |
| 14 | 2 | 60/203 | 5/10 | 110/198 | 43687 | 98/205 | 43687 | 72/205 | 43595 | 114/205 | 8/10 |
| 15 | 1 | 58/623 | 17/20 | 129/618 | 27/20 | 135/625 | 25/20 | 94/625 | 21/20 | 76/625 | 25/20 |
| 16 | 1 | 192/1501 | 14/35 | 456/1492 | 25/35 | 428/1503 | 24/35 | 271/1503 | 18/35 | 409/1503 | 23/35 |
| 18 | 1 | 138/1376 | 46/94 | 482/1375 | 73/94 | 288/1378 | 78/94 | 204/1378 | 61/94 | 469/1378 | 69/94 |
| 19 | 1 | 148/424 | 33/54 | 313/419 | 56/54 | 297/426 | 55/54 | 181/426 | 36/54 | 289/426 | 53/54 |
| 21 | 1 | 91/262 | 43699 | 125/261 | 43821 | 193/264 | 21/22 | 126/264 | 13/22 | 159/264 | 20/22 |
| 24 | 1 | 140/609 | 15/44 | 269/604 | 27/44 | 270/611 | 31/44 | 171/611 | 21/44 | 320/611 | 31/44 |
